# Microbiological Analyses of Subgingival Flora in Response to Probiotic Adjuncts to Non-surgical Periodontal Therapy: A Scoping Review

**DOI:** 10.7759/cureus.100604

**Published:** 2026-01-02

**Authors:** Bernis Aydin, Seray Z Ozturk, Emine Cifcibasi

**Affiliations:** 1 Periodontology, Istanbul University Institute of Health Sciences, Istanbul, TUR; 2 Periodontology, Istanbul University Faculty of Dentistry, Istanbul, TUR

**Keywords:** microbial analyses, non-surgical periodontal therapy, periodontitis, probiotics, subgingival microbiota

## Abstract

This scoping review aimed to map the subgingival microbiological analysis methods used in randomized controlled trials evaluating probiotics as adjuncts to non-surgical periodontal therapy (NSPT) and to summarize the microbial and clinical outcomes reported. A comprehensive search of PubMed, Scopus, and Web of Science from January 2008 to October 2025 identified nine eligible studies that employed culture-based techniques, PCR/qPCR, DNA-DNA hybridization, and 16S rRNA sequencing of subgingival plaque samples. Evidence suggests that probiotics may provide short-term, strain-dependent modulation of the microbiota, characterized by transient reductions in key periodontal pathogens and early improvements in inflammatory parameters. However, substantial heterogeneity in microbiological methods limits direct comparison across studies and contributes to uncertainty regarding long-term ecological and clinical effects. While probiotics appear to offer supportive benefits during the early healing phase following NSPT, persistent microbial stability, durable colonization, and sustained clinical advantages have not been conclusively demonstrated. Given the limited use of high-resolution sequencing techniques and marked methodological variability, current evidence remains insufficient to support routine clinical application. Future studies incorporating standardized ecological endpoints, extended follow-up periods, and advanced molecular analyses are essential to clarify the true clinical relevance of probiotic adjuncts in periodontal therapy.

## Introduction and background

Periodontal disease is a prevalent chronic infectious condition marked by inflammation of tooth-supporting tissues, progressive alveolar bone loss, and eventual tooth loss [1]. It is initiated by specific Gram-negative bacteria within the dental biofilm [2] and follows a chronic inflammatory course that may extend beyond local tissue destruction, as bacterial products and inflammatory mediators can enter the systemic circulation [3,4]. Accordingly, periodontitis should be regarded as a chronic infectious disease with systemic health implications [4].

Dental plaque develops as a structured biofilm in which salivary proteins facilitate bacterial adhesion and coordinated microbial activity [5]. This biofilm architecture enhances resistance to host immune responses by limiting immune cell penetration, reducing phagocytosis, and restricting antimicrobial diffusion [5,6]. Disruption of this ecological balance leads to dysbiosis, characterized by a shift from commensal microorganisms toward a pathogen-enriched biofilm dominated by Gram-negative anaerobes [7]. Socransky et al. [8] demonstrated that increasing proportions of red and orange complex species-including *Porphyromonas gingivalis*, *Tannerella forsythia*, and *Aggregatibacter actinomycetemcomitans*-parallel worsening clinical periodontal parameters, reflecting a disease-associated microbial profile. Notably, keystone pathogens such as *P. gingivalis* can subvert host immune defenses, sustaining inflammation while impairing microbial clearance [7], thereby promoting connective tissue destruction and alveolar bone resorption [8].

Given the pivotal role of dysbiosis in disease progression, non-surgical periodontal therapy (NSPT), which includes oral hygiene instruction and scaling and root planing (SRP), is considered the gold standard because it disrupts subgingival biofilm and facilitates partial microbial re-establishment [9]. However, anatomical complexities may restrict mechanical access, permitting pathogenic bacteria to persist [10]. Additionally, recolonization may restore subgingival microbial profiles to pretreatment levels within approximately 4-8 weeks, underscoring the necessity for adjunctive strategies to maintain clinical and microbiological improvements [11].

Adjunctive therapies, including antibiotics and antiseptics, have been extensively studied [12]. However, the European Federation of Periodontology (EFP) 2020 S3-level guidelines emphasize that the indications for systemic antibiotics have been significantly restricted [9]. The overuse of antibiotics contributes to antimicrobial resistance, which is a significant global health issue [13], and the microbiological benefits of these agents are often transient [14]. These challenges have led to increased interest in biologically compatible adjuncts, such as probiotics [15]. While the EFP 2020 guidelines reference probiotics for the first time and identify *Lactobacillus reuteri* strains as potentially beneficial, the current evidence base is insufficient to support their routine clinical application [9].

Probiotics, defined as “live microorganisms which, when administered in adequate amounts, confer a health benefit on the host” [16], have demonstrated potential in reducing pathogenic bacterial loads, improving periodontal parameters, and modulating inflammatory responses [17]. However, their effects differ significantly based on strain specificity, delivery method, dosage, and patient-related biological factors [15,16]. Additionally, heterogeneity in study designs and the limited oral colonization capacity of many strains complicate interpretation of the available evidence [15].

Accurate characterization of subgingival microbiota is critical for elucidating the effects of probiotics [18]. Traditional culture-based methods identify only a limited subset of oral microorganisms because of stringent growth requirements and low sensitivity [19]. Molecular techniques, including PCR, qPCR, and DNA-DNA hybridization, have enhanced the detection of specific species without the need for cultivation, but these approaches remain confined to predefined taxa [20]. The advent of next-generation sequencing (NGS), particularly 16S rRNA gene sequencing, has facilitated comprehensive, culture-independent profiling of complex microbial communities, encompassing previously uncultivable taxa [21]. More advanced approaches, such as shotgun metagenomics and metatranscriptomics, now enable functional and strain-level analyses [22], thereby supporting the development of targeted ecological therapies such as probiotics.

In light of these developments, the present scoping review aims to systematically evaluate the microbiological analyses performed in studies that have investigated the adjunctive use of probiotics in combination with NSPT, specifically those employing subgingival plaque samples for microbial assessment. By mapping the methodologies, sequencing approaches, and analytical strategies used to characterize subgingival microbiota in probiotic intervention studies, this review seeks to clarify how probiotics influence the composition of the microbial community in the periodontal environment. As a secondary objective, the review will also synthesize available evidence on the clinical outcomes associated with probiotic supplementation, providing an integrated overview of their potential therapeutic relevance and identifying key gaps to guide future research.

## Review

Materials and methods

Study Design

This scoping review was conducted following the methodological framework proposed by Reference [23] and subsequently refined by Reference [24], which provides a structured approach for mapping key concepts and evidence in emerging or heterogeneous research areas. The review was reported in accordance with the Preferred Reporting Items for Systematic Reviews and Meta-Analyses Extension for Scoping Reviews (PRISMA-ScR) guidelines to ensure transparency and methodological rigor [25]. A review protocol was developed a priori, defining the research question, search strategy, eligibility criteria, data charting variables, and overall synthesis approach.

PRISMA-ScR Flow Diagram

**Figure 1:**
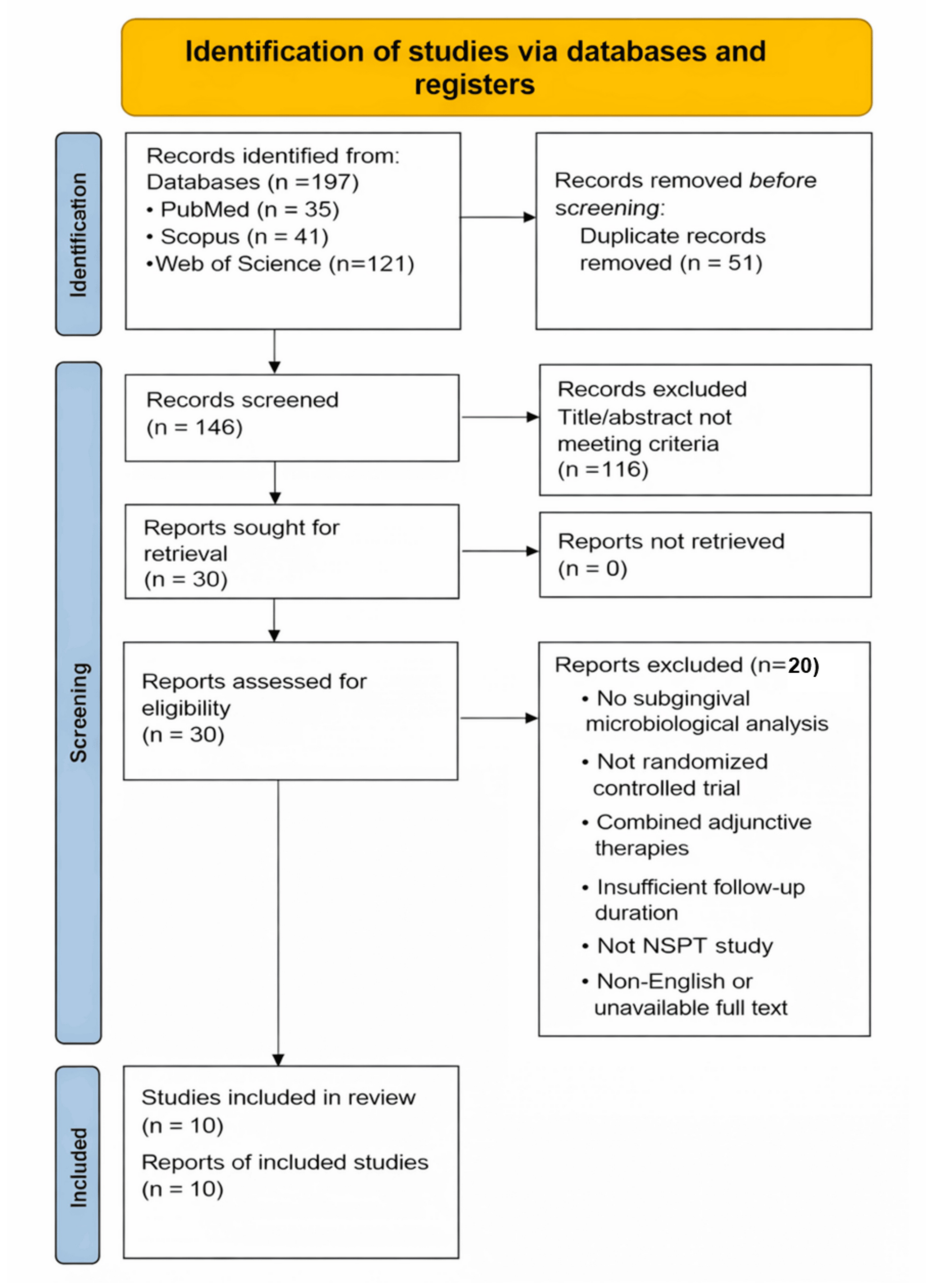
PRISMA 2020 flow diagram for study selection in the probiotic adjunctive NSPT scoping review (n = 10) PRISMA: Preferred Reporting Items for Systematic Reviews and Meta-Analyses; NSPT: non-surgical periodontal therapy

The PRISMA 2020 flow diagram [26] in Figure 1 outlines the identification, screening, eligibility assessment, and final inclusion process for the scoping review evaluating RCTs of probiotic supplementation as an adjunct to NSPT. A total of 197 records were identified across three databases (PubMed = 35, Scopus = 41, and Web of Science = 121). Following the removal of 51 duplicates, 146 records remained for title and abstract screening.

After screening, 30 full-text articles were assessed for eligibility. Of these, 20 articles were excluded for the following reasons: lack of subgingival microbiological analysis, non-randomized study design, use of combined adjunctive therapies, insufficient follow-up duration, not meeting NSPT criteria, or non-English/unavailable full text. Ultimately, 10 studies met the inclusion criteria and were included in the final scoping review synthesis.

Research Question

The review was guided by the following question: In patients with periodontitis undergoing NSPT, what microbiological methods have been used to assess subgingival microbiota when probiotics are provided as an adjunct, compared with NSPT alone or placebo, and what microbial outcomes have been reported in randomized controlled clinical trials?

Search Strategy

A comprehensive literature search was performed in PubMed/MEDLINE, Scopus, and Web of Science from January 2008 to October 2025. Searches were limited to English-language human studies. The following keyword and MeSH-based Boolean strategy was applied, with minor syntax adaptation per database: (periodontitis OR "periodontal disease") AND, (probiotic) AND, ("scaling and root planing" OR SRP OR "non-surgical periodontal therapy" OR NSPT) AND, (microbiome OR microbiota OR biofilm OR microbial OR PCR OR qPCR OR checkerboard OR "DNA-DNA hybridization" OR sequencing). Additionally, manual reference list screening was conducted to identify missed eligible studies.

Eligibility Criteria

Inclusion criteria: The inclusion criteria were restricted to randomized controlled clinical trials, either parallel-arm or split-mouth in design, involving human participants diagnosed with periodontitis. Eligible studies evaluated probiotics as adjuncts to NSPT, with or without accompanying oral hygiene instruction and SRP, and were published in English in peer-reviewed journals. Furthermore, studies were required to report subgingival microbiological outcomes using at least one recognized analytical method, including culture-based techniques, PCR or qPCR, DNA-DNA hybridization (checkerboard), 16S rRNA sequencing, or metagenomic approaches.

Exclusion criteria: The exclusion criteria encompassed all non-human and laboratory-based research, including animal studies and in vitro experiments, as well as observational designs, case reports, letters, and conference abstracts, and no review articles were considered. Studies in which probiotics were combined with other adjunctive therapeutic modalities-such as antibiotics, antiseptics, lasers, or photodynamic therapy-were excluded to isolate the specific effect of probiotic supplementation. Articles were also removed if they lacked subgingival microbiological analysis, were unavailable in full text, or were published in languages other than English.

Screening Process

Study selection was conducted by two independent reviewers (Bernis Aydin and Emine Cifcibasi), who screened all titles, abstracts, and full-text articles in accordance with the predefined eligibility criteria. Each reviewer completed the screening independently using a standardized form to minimize selection bias. Any discrepancies or uncertainties regarding study inclusion were first discussed between the two reviewers. When consensus could not be reached, a third reviewer (Seray Z. Ozturk) was consulted to provide an additional assessment and finalize the decision.

The overall selection process adhered strictly to the PRISMA-ScR framework, which includes four sequential stages: identification, screening, eligibility, and inclusion. All decisions made throughout the process were documented, and the final study selection is illustrated in the PRISMA-ScR flow diagram.

Data Charting

Data from the included studies were extracted using a standardized charting form to ensure consistency and minimize reporting bias. Extracted variables encompassed the author, year, and country of each study; study design and sample size; participant characteristics and periodontal diagnosis; and detailed information on the probiotic intervention, including strain(s), formulation, dosage, administration route, and duration. Additionally, data regarding the NSPT protocol, subgingival sampling method, microbiological analysis technique, and follow-up duration were collected. Key clinical and microbiological outcomes reported in each study were also systematically recorded. Owing to the substantial methodological heterogeneity across trials, the extracted data were synthesized descriptively rather than quantitatively. Consistent with the methodological framework of scoping reviews, a formal risk of bias assessment was not performed. Instead, potential methodological limitations and sources of bias were descriptively considered during data synthesis and interpretation to provide appropriate contextualization of the findings.

Ethical Considerations

As this review used previously published data, no ethical approval was required.

Measurement Tools and Licensing Information

All methodological tools, diagnostic frameworks, and clinical indices referenced in this review are standard instruments that are freely accessible for academic and non-commercial use. None of the tools utilized in the included studies requires formal licensing, purchase, or special authorization. All instruments have been previously published in the scientific literature and are reproducible with appropriate citation. No copyrighted or proprietary measurement systems were employed in the preparation of this review.

Results

An overview of the included randomized controlled trials (RCTs) is presented in Table 1.

**Table 1:** Overview of included randomized controlled trials PD: probing depth; GI: gingival index; TVC: total viable cell count; CAL: clinical attachment level; SRP: scaling and root planning; CFU: colony-forming unit; Aa: *Aggregatibacter actinomycetemcomitans*; Pg: *Porphyromonas gingivalis*; Pi: *Prevotella intermedia*; PI: plaque index; SBI: sulcus bleeding index; MALDI-TOF: matrix-assisted laser desorption/ionization time-of-flight; Tf: *Tannerella forsythia*; Fn: *Fusobacterium nucleatum*; BoP: bleeding on probing; PPD: probing pocket depth; BI: bleeding index; GCF: gingival crevicular fluid; SI: subgingival instrumentation; NSPT: non-surgical periodontal therapy; SMDI: subgingival microbial dysbiosis index; ANOSIM: analysis of similarities

Study (year)	Sample size (n)	Periodontal diagnosis	Probiotic strain	Delivery & duration	Microbiological method	Subgingival microbial outcomes	Clinical outcomes	Other outcomes	Follow-up duration
Tekce et al. (2015) [27]	40 (20 probiotic and 20 placebo)	Chronic periodontitis; ≥2 teeth with PD 5–7 mm and GI ≥ 2	*Lactobacillus reuteri* (Prodentis)	Lozenge, twice daily for 3 weeks	Culture-based subgingival analysis (TVC & obligate anaerobes)	TVC ↓; obligate anaerobes at days 21, 90, and 180 ↓ return to baseline at day 360	Greater PD and CAL improvements vs. placebo at all time points	No non-subgingival samples analyzed	360 days (1 year)
Vivekananda et al. (2010) [28]	30 (10 SRP + probiotic, 10 probiotic, and 10 SRP + placebo)	Chronic generalized periodontitis; mild-moderate pockets (5–7 mm)	*L. reuteri* (DSM 17938 + ATCC PTA 5289)	Lozenge, twice daily from day 21–42 (21 days)	Culture-based CFU counts (Aa, Pg, Pi)	Aa, Pg, Pi ↓ by ~1 log10; strongest reduction with SRP + Prodentis	SRP + probiotic group showed the greatest reductions in PI, GI, SBI, PD, and CAL (PD ≈1.3 mm, CAL ≈1.1 mm)	No non-subgingival samples analyzed	42 days
Pudgar et al. (2021) [29]	40 (20 probiotic and 20 placebo)	Untreated advanced periodontitis (stage III–IV, 2018 classification); PD ≥ 5 mm on ≥4 teeth in ≥4 quadrants	*Lactobacillus brevis* + *Lactobacillus plantarum*	Local gel after SRP + daily lozenges for 3 months	Culture-based; MALDI-TOF identification	Placebo showed ↓ Pg/Tf; probiotics only ↑ suppression of Fn with no major pathogen reduction	Similar PD/CAL improvements; probiotics improved bleeding but had more residual diseased sites (OR = 2.12)	No non-subgingival samples analyzed	3 months
Teughels et al. (2013) [30]	30 (15 probiotic and 15 placebo)	Moderate to severe chronic periodontitis	*L. reuteri* (Prodentis) (DSM 17938 & ATCC PTA5289)	Lozenge twice daily for 12 weeks, starting immediately after SRP	qPCR analysis (Pg, Tf, Aa, Pi, Fn, total bacterial load)	Pg ↓ significantly more in the probiotic group; Tf, Aa, Pi, Fn ↓ in both groups; total load ↓ without intergroup difference	Greater PD/CAL improvement gain in moderate/deep pockets; fewer sites needing surgery; BoP ↓ in both groups	No other biological materials analyzed	12 weeks
Laleman et al. (2015) [31]	48 (24 probiotic and 24 placebo)	Untreated moderate-to-severe chronic periodontitis	*Streptococcus oralis* KJ3, *Streptococcus uberis* KJ2, *Streptococcus rattus* JH145	Oral tablets twice daily for 12 weeks after SRP	qPCR analysis (Pg, Tf, Aa, Pi, Fn, total bacterial load) qPCR-based subgingival analysis (Pg, Tf, Pi, Fn)	No significant intergroup differences; Fn showed a non-significant reduction in the probiotic group (p = 0.06)	Both groups showed similar PD, CAL, BoP, and GI improvements; only plaque-positive sites were significantly lower in the probiotic group at week 24	Salivary Pi significantly decreased in the probiotic group at week 12; no other biological samples analyzed	24 weeks
Laleman et al. (2019) [32]	39 (19 probiotic and 20 control)	Residual pockets after initial non-surgical periodontal therapy (≥6 mm or 5 mm + BoP)	*L. reuteri* (ATCC PTA 5289 & DSM 17938)	Lozenges twice daily for 12 weeks after re-instrumentation	qPCR-based subgingival analysis of periodontopathogens (Pg, Pi, Aa, Fn)	No significant intergroup differences in subgingival pathogen levels by qPCR	Greater PPD reduction, more pocket closure, and fewer sites needing surgery in the probiotic group	Saliva, tongue, and supragingival qPCR analyses performed; no significant microbial changes	24 weeks
Vanditha et al. (2025) [33]	30 patients (60 sites): 30 sites: SRP + probiotic fiber; 30 sites: SRP + placebo fiber	Chronic periodontitis (residual pockets ≥ 5 mm)	L. reuteri	Locally delivered biodegradable probiotic fiber; single application adjunctive to SRP	qRT-PCR targeting Pg	Significant reduction in Pg load in test sites compared with control	Improvements in PI, ≈58% in BI, and ≈43% in both PPD and CAL in both groups; no significant intergroup clinical differences	No non-subgingival samples analyzed	3 months
Invernici et al. (2018) [34]	41 (20 probiotic and 21 control)	Generalized chronic periodontitis (≥30% sites with PPD ≥ 4 mm & CAL ≥4 mm)	*Bifidobacterium animalis* subsp. *lactis* HN019	Lozenges twice daily for 30 days after SRP	Checkerboard DNA-DNA hybridization (40 taxa) + qPCR for *B. lactis* HN019 (subgingival)	Red/orange complexes ↓; blue complex ↑ vs. control; *B. lactis* HN019 DNA detected only in the probiotic group at 30 & 90 days	The probiotic group showed greater PPD reduction (−3.52 mm), CAL gain (−3.45 mm), and BoP reduction compared with control	GCF: IL-1β and IL-8 lower in the probiotic group; IL-10 increased only in the probiotic group at 30 days	90 days (baseline, 30 & 90 days)
de Oliveira et al. (2022) [35]	42 (19 probiotic and 23 control)	Untreated periodontitis (stage III/IV, grade B/C)	Multistrain probiotic (5 *Lactobacillus* spp. + 3 *Bifidobacterium* spp.)	Lozenges twice daily for 30 days after SRP. Oral capsules once daily for 30 days during 4-week subgingival instrumentation	Checkerboard DNA-DNA hybridization (subgingival); 16S rRNA sequencing (stool)	Most subgingival species decreased post-SI in both groups; the probiotic group had more species with reduction but no significant intergroup differences at 2 months	Both groups showed significant improvements in PD, CAL, and BoP; deep pockets improved markedly; poor responders more common in placebo	Gut 16S analysis showed shifts in *Proteobacteria*, *Actinobacteria*, *Bacteroidetes*, and *Verrucomicrobia*, with oral-gut microbial clusters linked to response. Soft-stool events were more common in the probiotic group	60 days (2 months)
Huo et al. (2025) [36]	40 (20 probiotic and 20 control)	Stage III-IV periodontitis (2018 classification) in systemically healthy adults	*Limosilactobacillus reuteri* (DSM 17938 & ATCC PTA 5289)	Oral probiotics twice daily (1 × 10^8^ CFU/strain) for 21 days after full-mouth NSPT; reviews at 1, 3, and 6 months	16S rRNA gene sequencing (DADA2 pipeline) of subgingival biofilm (amplicon sequencing variants, SMDI)	α-Diversity unchanged between groups; ANOSIM showed significant compositional differences; the test group showed greater SMDI reduction and decreases in Tf and *Streptococcus constellatus* at 1 month, indicating a shift toward a less dysbiotic subgingival microbiota	Both groups showed significant improvements in PD, CAL, BI, and pocket-depth distribution; the probiotic group showed additional benefit on CAL and medium pocket reduction at 6 months compared with NSPT alone	Salivary 16S rRNA sequencing: similar α-diversity between groups; some taxa (e.g., *Prevotella nanceiensis*) changed over time; salivary SMDI decreased after therapy, with patterns paralleling subgingival changes	6 months

Microbiological Outcomes

Culture-based microbiological findings: Across culture-based studies, probiotic supplementation consistently demonstrated a modulatory effect on the subgingival ecosystem. Tekce et al. [27] showed that adjunctive *L. reuteri* lozenges with SRP produced greater reductions in total viable counts and the proportion of obligate anaerobes at 21, 90, and 180 days compared with placebo, with transient subgingival colonization detected at days 21 and 90.

In Vivekananda et al. [28], groups receiving *L. reuteri*-either alone or combined with SRP-exhibited significantly greater reductions in key periodontal pathogens (*A. actinomycetemcomitans*, *P. gingivalis*, *Prevotella intermedia*, and *Fusobacterium nucleatum*) than both baseline and SRP + placebo. The SRP + probiotic group achieved the most extensive microbial suppression, while probiotic use alone reduced pathogen counts more effectively than SRP alone.

Pudgar et al. [29] reported that *Lactobacillus brevis* (CECT7480) and *Lactobacillus plantarum* (CECT7481) produced downward trends in total anaerobes and cultivable pathogens, although these changes were not statistically significant compared with placebo. Nonetheless, more consistent reductions in organisms such as *P. gingivalis* and *P. intermedia* suggested modest, strain-specific antimicrobial benefits.

PCR/qPCR-based microbiological findings: Across RCTs using PCR-based methods, the microbiological effects of probiotic supplementation were variable and largely species-specific. In Teughels et al. [30], *L. reuteri* produced its most notable impact on *P. gingivalis*, with significantly greater reductions in the probiotic group at weeks 9 and 12. Although all pathogens declined following SRP, intergroup differences for *A. actinomycetemcomitans*, *F. nucleatum*, *T. forsythia*, and *P. intermedia* in subgingival plaque were not significant. Broader probiotic-associated decreases were observed in supragingival plaque and saliva, particularly for *P. gingivalis*, with a smaller adjunctive effect for *P. intermedia* in saliva at week 12.

In Laleman et al. [31], major periodontal pathogens showed substantial reductions in both groups over 24 weeks, without significant intergroup differences; the only probiotic-specific finding was a significant reduction in *P. intermedia* in saliva at 12 weeks. Laleman et al. [32] similarly reported that SRP drove marked decreases in all evaluated pathogens, with no probiotic-related intergroup differences. Culture analysis confirmed that the administered *L. reuteri* strains did not colonize periodontal pockets.

In Vanditha et al. [33], microbiological analysis using qRT-PCR demonstrated a significant reduction in *P. gingivalis* levels from baseline to three months in both groups. Although the probiotic group showed a greater decrease in bacterial load, no statistically significant intergroup difference was observed.

DNA-DNA hybridization-based microbiological findings: Checkerboard DNA-DNA hybridization studies have shown that probiotic supplementation can modulate the subgingival microbiota by suppressing periodontal pathogens and enhancing health-associated species. Invernici et al. [34] reported more pronounced reductions-at 90 days-in red complex bacteria such as *P. gingivalis* and *Treponema denticola*, as well as orange complex species including *F. nucleatum vincentii*, *Campylobacter showae*, and *Eubacterium nodatum* in the probiotic group, along with increases in commensals such as *Actinomyces​​​​​​​ naeslundii* and *Streptococcus*​​​​​​​* mitis*. At the microbial-complex level, the probiotic group exhibited lower proportions of the orange complex at 30 days and the red complex at 90 days, whereas blue complex levels were significantly elevated at 90 days. qPCR analysis confirmed short-term integration of *Bifidobacterium lactis* HN019, with increased DNA copy numbers at 30 and 90 days only in the probiotic group, supporting transient colonization.

In contrast, de Oliveira et al. [35] observed significant reductions in subgingival microbial diversity and decreases across multiple periodontal taxa in both probiotic and placebo groups, reflecting a general shift toward a healthier microbiota following therapy. Although more species decreased in the probiotic arm, no statistically significant intergroup differences were found for individual taxa. Additionally, gut microbiome analysis via 16S rRNA sequencing revealed several phylum- and genus-level changes within both groups, but no consistent probiotic-specific effects. Overall, systemic probiotic supplementation did not demonstrate clear additive changes in subgingival or gastrointestinal microbial communities beyond those achieved through mechanical therapy.

16S rRNA sequencing-based microbiological findings: Evidence from 16S rRNA sequencing remains limited, with only one RCT available. In the study by Huo et al. [36], adjunctive *L. reuteri* produced rapid and distinct microbiological shifts within the subgingival ecosystem. At one month, *T. forsythia*-a key red-complex pathogen-decreased significantly only in the probiotic group, accompanied by an early reduction in *Streptococcus constellatus*, a species linked to refractory periodontitis. *Prevotella saccharolytica* showed a significant post-treatment increase in the probiotic group, although its clinical significance remains uncertain.

Linear discriminant analysis effect size (LEfSe) analysis demonstrated the greatest group separation at one month, with enrichment of *Bacteroidota*, *Prevotellaceae*, *Phascolarctobacterium*, and *Collinsella* in the probiotic group, whereas *Paenibacillaceae *and *Lactobacillaceae* were enriched in controls. The subgingival microbial dysbiosis index decreased in both groups after therapy but showed a more pronounced early improvement with probiotics, indicating a transient shift toward a less dysbiotic profile. However, these effects diminished by six months, suggesting that sustained or repeated probiotic administration may be required to maintain early microbiological benefits.

Clinical Outcomes

Across the included RCTs, adjunctive probiotic therapy-most commonly *L. reuteri*, but also *B. lactis* HN019, *L. brevis* CECT7480, *L. plantarum* CECT7481, and multistrain systemic formulations-generally supported clinical periodontal improvement in patients with stage II-IV periodontitis, although the magnitude and consistency of benefit varied across studies. Several trials demonstrated clear clinical advantages for probiotic-treated groups. Tekce et al. [27] reported significantly lower plaque index (PI), gingival index (GI), bleeding on probing (BoP), and probing depth (PD) scores at all follow-ups-including at one year-and fewer residual deep pockets and reduced surgical need, while Vivekananda et al. [28] showed that SRP combined with *L. reuteri* produced the greatest reductions in PD clinical attachment level (CAL), with even probiotic use alone outperforming SRP + placebo for PI, GI, and bleeding outcomes. Similarly, Teughels et al. [30] observed greater PD reduction and CAL gain in initially moderate and deep pockets, fewer residual sites ≥ 5 mm, marked BoP reduction, and more favorable Lang and Tonetti risk profiles at 12 weeks in the *L. reuteri* group.

Invernici et al. [34] further demonstrated that *B. lactis* HN019 supplementation led to significantly greater probing pocket depth (PPD) reduction and CAL gain in deep pockets at 90 days, with 58% of deep sites resolving to ≤3 mm compared with 22% in controls. Other studies found clinical outcomes to improve substantially after SRP but without additional benefit from probiotics. Both Laleman et al. trials [31,32] showed parallel reductions in PPD across shallow, moderate, and deep sites, similar CAL gains, and comparable decreases in BoP, plaque indices, and surgical need by 24 weeks, with only a modest improvement in plaque percentage at 24 weeks in the probiotic arm of the 2015 trial.

Vanditha et al. [33] demonstrated significant intragroup clinical improvements from baseline to three months, with reductions of 45.66% in PI, bleeding index (BI), PPD, and CAL in test and control sites; however, no statistically significant intergroup differences were observed for these clinical parameters. Pudgar et al. [29] also reported similar improvements in PD, CAL, BoP, and plaque levels in both groups, although probiotics yielded significantly greater reductions in gingival bleeding; however, diseased sites (PPD > 4 mm + BoP) were less likely to resolve in the probiotic group. Systemic multistrain supplementation in the de Oliveira et al. trial [35] did not provide additional short-term clinical benefits, despite substantial improvements in both groups following subgingival instrumentation. In the only available 16S rRNA-based study, Huo et al. [36] found no early (one month) clinical advantage for *L. reuteri*, but a delayed benefit emerged: by six months, the probiotic group exhibited significantly greater CAL gain and resolution of medium-depth pockets, indicating enhanced stability of clinical healing over time. Collectively, while SRP remained the primary driver of periodontal improvement across all studies, several trials-particularly those employing *L. reuteri* or *B. lactis* in patients with moderate to advanced disease-demonstrated meaningful adjunctive clinical benefits, especially in reducing bleeding, promoting PD reduction and CAL gain in deeper sites, and improving overall risk profiles.

Discussion

The present scoping review aimed to map and critically examine the microbiological approaches used to assess the potential effects of probiotic adjuncts on the subgingival ecological recovery trajectory-a key determinant of long-term periodontal stability. The consistent inclusion of subgingival plaque sampling across all eligible trials strengthens the relevance of this synthesis, as the periodontal pocket represents the primary pathogenic niche. By systematically cataloguing and comparing the microbiological methodologies applied, this review provides a methodological framework to support more robust interpretation of probiotic-related microbial outcomes and to inform the design of future clinical trials.

When interpreted in the context of existing literature, this focus underscores an important methodological gap. Recent systematic reviews and meta-analyses evaluating the adjunctive use of probiotics with NSPT have predominantly emphasized clinical outcomes such as PPD reduction, clinical attachment gain, and bleeding indices [37,38]. Although microbiological findings have been reported, they have frequently been derived from heterogeneous datasets, with limited critical appraisal of the analytical methodologies applied [39,40]. To date, no review has systematically mapped and critically examined the microbiological analysis techniques used across RCTs in this field.

Moreover, most included trials relied on closed-ended, targeted microbiological methods-primarily species-specific qPCR or checkerboard DNA-DNA hybridization-focused on a limited set of predefined periodontal pathogens. While these approaches are highly sensitive for selected taxa, they inherently constrain ecological interpretation by failing to capture broader community-level shifts and recolonization dynamics. In contrast, open-ended sequencing-based techniques, such as 16S rRNA gene sequencing, remain markedly underutilized in probiotic adjunct trials, thereby limiting insight into dysbiosis modulation and strain-specific ecological effects.

Recent Evidence: Microbiological Approaches

Microbiological assessment of heterogeneity and its implications: A key observation across the included studies was the considerable methodological heterogeneity in microbiological assessment, encompassing traditional culture-based quantification [27-29], targeted pathogen-specific PCR/qPCR assays [30-33], DNA-DNA checkerboard hybridization enabling multiplex detection of established periodontal pathogens [34,35], and a single trial employing NGS via 16S rRNA gene amplicon analysis [36]. Although this diversity allows complementary insights into probiotic-microbiome interactions, it substantially constrains cross-study comparability and limits the synthesis of microbiological outcomes, reflecting a broader and well-recognized challenge within periodontal microbiome research.

Culture-based studies: historically valuable yet ecologically constrained: Early RCTs employing cultivation-based methodologies reported reductions in classical periodontal pathogens such as *P. gingivalis*, *A. actinomycetemcomitans*, and *P. intermedia* following probiotic administration [27-29]. While these observations initially substantiated the conceptual premise that probiotics may exert pathogen-suppressive effects, contemporary microbiological frameworks highlight critical limitations inherent to culture-based techniques. A substantial proportion of anaerobic, slow-growing, and stress-adapted subgingival taxa remain non-culturable under standard laboratory conditions [19], leading to systematic underestimation of the true microbial diversity, ecological resilience, and interspecies network dynamics characteristic of periodontal biofilms. Moreover, colony-forming unit quantification offers no insight into whether observed reductions represent transient microbial suppression or a more durable ecological reorganization-an essential distinction given that periodontal disease recurrence is strongly linked to long-term stability of the subgingival microbiome [41].

PCR/qPCR: sensitive but narrow in ecological scope: Most PCR/qPCR-based trials reported reductions or downward trends in target periodontal pathogens following probiotic supplementation. Although statistically significant changes were observed in some studies, intergroup differences were not consistently significant, and overall findings remained heterogeneous across trials [30-33]. Although these techniques offer high analytical sensitivity and robust quantitative precision, they inherently rely on the assumption that decreases in a limited panel of “key pathogens” reflect meaningful ecological improvement. This assumption is increasingly disputed, as contemporary microbial ecology research characterizes periodontitis as a polymicrobial dysbiotic condition rather than a classical monopathogenic infection [42]. Consequently, while qPCR effectively verifies biological activity and pathogen suppression, its ecological interpretability remains constrained: it offers no information on overall microbial diversity, commensal repopulation, keystone species behavior, or the structural and functional dynamics of microbial interaction networks-factors that are central to periodontal stability and long-term treatment success.

Checkerboard hybridization: broader coverage but closed-ended: Checkerboard assays, which quantify 40 subgingival taxa, offer greater breadth than single-target qPCR [34,35]. Although this represents a substantially broader analytical spectrum than single-target PCR/qPCR assays, the technique remains inherently constrained by its closed-ended design, as only taxa included in the probe panel can be detected. Consequently, checkerboard analysis provides limited insight into the broader ecological configuration of the subgingival microbiome, including shifts in low-abundance commensals, symbiotic network restoration, functional redundancy, and neutral drift dynamics [8,43].

16S rRNA sequencing: essential but underutilized: Only one RCT employed 16S rRNA sequencing [36], reporting changes in diversity metrics and dysbiosis indices. These findings suggest that probiotics may influence microbial ecology in ways that extend beyond simple pathogen suppression. The scarcity of sequencing-based investigations underscores a significant gap in both knowledge translation and methodological adoption: despite the widespread recognition of periodontitis as a dysbiosis-driven condition [43,44], most clinical trials continue to rely on conventional pathogen-centric assays, thereby constraining ecological interpretation.

Collectively, these observations indicate that the inconsistencies within the current evidence base stem primarily from methodological limitations rather than true biological discordance. The absence of standardized, ecology-oriented microbiological endpoints prevents definitive conclusions regarding whether probiotics merely reduce selected periodontal pathogens. It also limits the assessment of their potential influence on broader community-level features of the subgingival microbiota.

To address these interpretive limitations and improve mechanistic understanding, metagenomic approaches offer significant advantages. Shotgun metagenomic investigations in periodontal health and disease have revealed substantial differences not only in the taxonomic configuration of the subgingival microbiome but also in its functional gene repertoire, virulence determinants, and metabolic pathways [45,46]. Such high-resolution, function-oriented analyses facilitate the elucidation of molecular mechanisms related to colonization resistance, adhesion dynamics, and biofilm interactions, thereby informing the development of future mechanism-based, standardized probiotic protocols and enhancing the interpretability of probiotic effects within complex periodontal ecosystems.

Mechanism

Probiotics are proposed to exert their biological effects via multiple convergent mechanisms, including competitive exclusion of opportunistic pathogens, reinforcement of epithelial barrier integrity, and modulation of local and systemic immune responses. Evidence from experimental and gastrointestinal clinical studies consistently demonstrates that probiotics compete with pathogens for adhesion sites and nutrients, enhance barrier function, produce antimicrobial metabolites, and shift ecological networks toward a more balanced configuration [47,48]. Similar mechanisms have been observed on oral mucosal surfaces and within periodontal pockets, suggesting cross-anatomical parallels in host-microbe interactions [15]. However, variations in probiotic strain, dosage, delivery method, and follow-up duration complicate the interpretation of clinical and microbiological outcomes [39,49,50]. Current evidence indicates that probiotic efficacy is strain-dependent. For instance,* L. reuteri* consistently demonstrates improved pathogen suppression and clinical outcomes, likely attributable to its antimicrobial properties and colonization capacity [28,30,33,36]. In comparison, *L. brevis* and *L. plantarum* exhibit more variable or modest effects, occasionally resulting in increased residual diseased sites [29].

The mode of delivery represents an additional determinant of efficacy. For instance, certain studies compare strains administered as lozenges or chewable tablets, which may promote extended oral residence and mucosal adherence, while toothpaste-based formulations typically result in only brief exposure [27,51]. Moreover, limited research has assessed the persistence of microbial colonization, which is a critical criterion for establishing mechanistic validity.

Clinical Applications

Probiotics function as short-term ecological modulators following NSPT, contributing to reductions in inflammation-associated clinical parameters and targeted pathogens within four to 12 weeks [27-36]. However, the long-term ecological durability of these effects remains uncertain, as follow-up beyond six months was uncommon, and no study demonstrated sustained reprogramming of the subgingival microbiome.

Substantial differences in clinical and microbiological responses among individuals likely reflect variations in baseline microbial ecosystems, immune-metabolic profiles, periodontal stage and grade, and patient-specific factors such as smoking and diabetes [52,53]. These results are consistent with precision-medicine principles, which emphasize the importance of host-microbe interactions and the need for standardized, stage- and grade-based patient selection in future trials to reduce heterogeneity. Furthermore, several studies [27-33] included in this analysis were conducted prior to the 2017 periodontal classification [52] and therefore did not employ stage- and grade-based diagnostic criteria. The absence of a unified diagnostic framework hindered consistent patient characterization and likely contributed to increased variability in probiotic responses.

Periodontal pathogenesis is characterized by a self-sustaining inflammation-dysbiosis cycle [54], which creates an environment conducive to disease persistence. Evidence indicates that the stage and grade categories established in the 2017 classification correspond to distinct subgingival microbial signatures [52,55,56], suggesting a direct association between disease severity and microbial profiles. Consequently, probiotic responsiveness may differ according to disease severity, as specific microbial signatures may exhibit variable responses to intervention. This variability likely contributes to the heterogeneity observed in RCTs. Some studies reported notable clinical improvements with probiotic supplementation [27,28,30]; others have reported comparable outcomes between probiotic and control groups [29,31,35], particularly in trials lacking participant stratification by disease characteristics or employing pre-2017 classifications. Such inconsistencies in recruitment and classification methods contribute to the heterogeneous findings prevalent in current probiotic research.

Future Research Priorities

Future RCTs investigating probiotic-mediated periodontal microbiome modulation should implement NGS-based or multiomic platforms including metatranscriptomics, metabolomics, and culturomics; monitor strain survival, colonization, and clearance; assess ≥6-12-month ecological and clinical durability; integrate phenotype- and risk-based patient stratification; evaluate drug-microbiome and diet-microbiome interactions; investigate optimal dosing, timing, and delivery systems; and explore precision-strain synbiotic strategies targeting metabolic niches.

## Conclusions

Current evidence indicates that probiotics may offer short-term, strain-specific ecological modulation when used as adjuncts to NSPT. Multiple randomized trials have reported temporary reductions in key periodontal pathogens and early improvements in inflammation-related clinical parameters, suggesting a potential supportive role during the initial healing phase. However, durable microbial stabilization, long-term colonization, and sustained clinical benefits have not been conclusively established. Significant methodological heterogeneity, particularly in microbiological sampling and analytical techniques, as well as the limited application of high-resolution sequencing methods, continue to limit the comparability and interpretability of results. Therefore, routine clinical adoption of probiotic adjuncts is not yet justified. More rigorous RCTs with standardized ecological endpoints, extended follow-up durations, and advanced microbiome analyses are necessary to elucidate the mechanistic and clinical significance of probiotic supplementation in periodontal therapy.
